# How West African countries prioritize health

**DOI:** 10.1186/s41182-021-00380-6

**Published:** 2021-10-26

**Authors:** Yusuff Adebayo Adebisi, Aishat Alaran, Abubakar Badmos, Adeola Oluwaseyi Bamisaiye, Nzeribe Emmanuella, Alison Ubong Etukakpan, Iyiola Olatunji Oladunjoye, Oladipo Oluwaseyifunmi, Shingin Kovona Musa, Temiwunmi Akinmuleya, Omotayo Carolyn Olaoye, Obafemi Arinola Olarewaju, Don Eliseo Lucero-Prisno

**Affiliations:** 1 African Young Leaders for Global Health, Abuja, Nigeria; 2 Global Health Focus, Kigali, Rwanda; 3 Faculty of Pharmacy, University of Ibadan, Ibadan, Nigeria; 4 Faculty of Pharmaceutical Sciences, University of Ilorin, Ilorin, Nigeria; 5 National Primary Healthcare Development Agency, Abuja, Nigeria; 6 Centre for Tropical Medicine and Global Health, University of Oxford, Oxford, UK; 7 Department of Child Health, Tamale Teaching Hospital, Tamale, Ghana; 8 Faculty of Pharmaceutical Sciences, University of Uyo, Uyo, Nigeria; 9 Department of Microbiology, University of Ilorin, Ilorin, Nigeria; 10 Faculty of Public Health, College of Medicine, University of Ibadan, Ibadan, Nigeria; 11 Faculty of Pharmaceutical Sciences, Ahmadu Bello University, Zaria, Nigeria; 12 College of Medical, Veterinary and Life Sciences, University of Glasgow, Glasgow, UK; 13 Force Headquarters, Police Medical Services, Falomo Hospital, Ikoyi, Lagos, Nigeria; 14 Department of Global Health and Development, London School of Hygiene and Tropical Medicine, London, UK

**Keywords:** Health financing, Health systems, Universal Health Coverage, ECOWAS, West Africa

## Abstract

**Background:**

The goal of Universal Health Coverage (UHC) is to ensure that everyone is able to obtain the health services they need without suffering financial hardship. UHC remains a mirage if government health expenditure is not improved. Health priority refers to general government health expenditure as a percentage of general government expenditure. It indicates the priority of the government to spend on healthcare from its domestic public resources. Our study aimed to assess health priorities in the Economic Community of West African States (ECOWAS) using the health priority index from the WHO’s Global Health Expenditure Database.

**Method:**

We extracted and analysed data on health priority in the WHO’s Global Health Expenditure Database across the 15 members of the ECOWAS (Benin, Burkina Faso, Cabo Verde, Cote d'Ivoire, The Gambia, Ghana, Guinea, Guinea-Bissau, Liberia, Mali, Niger, Nigeria, Senegal, Sierra Leone, and Togo) from 2010 to 2018 to assess how these countries prioritize health. The data are presented using descriptive statistics.

**Results:**

Our findings revealed that no West African country beats the cutoff of a minimum of 15% health priority index. Ghana (8.43%), Carbo Verde (8.29%), and Burkina Faso (7.60%) were the top three countries with the highest average health priority index, while Guinea (3.05%), Liberia (3.46%), and Guinea-Bissau (3.56%) had the lowest average health priority in the West African region within the period of our analysis (2010 to 2018).

**Conclusion:**

Our study reiterates the need for West African governments and other relevant stakeholders to prioritize health in their political agenda towards achieving UHC.

## Introduction

Health systems in Sub-Saharan Africa including West Africa have long been plagued with a double burden of communicable and non-communicable diseases [1] and presently, the COVID-19 pandemic [2]. Despite the advancement in health across the world, the region continues to lag behind. There is at least a decade difference in the life expectancies in the African region compared to other regions of the world. This is in part due to the weak healthcare systems [3]. Although the global maternal mortality rate fell by 44% between 1990 and 2015, 99% of the global maternal mortality rate is now being accounted for in the developing world with over 66% of the cases being reported in West Africa and other sub-Saharan African countries [4]. The infant and under-five mortality rates are also comparable; while there have been a massive decline in the world under-five mortality rates (59% decrease from 1990 to 2019), the statistic is not very promising in sub-Saharan Africa, as the region accounts for the highest under-five mortality rates in the world [5, 6]. One in 13 children in the region dies before the age of 5 [5]. The HIV epidemic also continues to disproportionally affect the region, with insufficient antiretroviral treatment and AIDS-related complications playing a huge role in infant mortality and reduced life expectancy [7, 8].

This huge disparity and the persistence of ill-health in the region are not due to the dearth of initiatives or policies to reform the healthcare system. Every decade, since the 1940s has seen health policymakers, professionals, and providers launch global and national initiatives to address the growing health challenges and needs of people living in sub-Saharan Africa. However, only a few have had any success [1]. The Abuja Declaration made in 2001 remains one of the most significant, where the government of the African Union countries met and pledged to allocate at least 15% of their budget to health to address the massive burden of health problems facing countries in Africa, particularly within the context of a growing burden of HIV, AIDS, TB, and malaria [9]. However, two decades after the declaration, only three countries were listed as making progress towards the pledge [3, 10]. The countries are Rwanda, Botswana, and Zambia, with no countries in West Africa. More recently, the high-level task force on Innovative Financing for Health system (HLTF) recommended that low-income countries needed at least 44 USD per capital to deliver an essential package of health services [3, 11]. For sub-Saharan Africa to meet the target of the Universal Health Coverage (UHC) and Sustainable Development Goals (SDGs) by 2030, the countries need to increase the government general health expenditure from an average of 5.6% to 7.5%, and this would require at least 371 billion US dollars yearly to bridge the gap [12].

Despite the benefits health insurance has reaped for healthcare in many high-income countries, African countries including West African countries still struggle with the implementation and sustainability of effective and efficient health insurance systems [3]. A protocol for systematic review revealed that even though there are numerous studies on the impact of health insurance on access to medical services, few have considered West Africa as a whole [6]. Despite many years of investments and targeted work by the government and private insurance establishments, West African countries continue to suffer from significant out-of-pocket payment for healthcare [3]. While it has never been more critical for governments in the region to be more committed to improve the healthcare status of their people and meet international goals, efforts towards this remain grossly insufficient. West African government needs to prioritize healthcare for several reasons. First, health is a human right that every citizen of every country should have access to. Second, good health is a prerequisite to the human development and economic growth of any country [13]. Third, national health systems in West Africa are underfunded and the adequate manpower to efficiently run the healthcare system is not available [14, 15]. In addition, healthcare system readiness and proper functioning are important in combating epidemics and pandemics. The government’s commitment to healthcare in this region has been a subject under constant scrutiny, because even if there is a fixed target for the amount government spends on health, there is a need for robust data and monitoring of government health spending. The levels of government health expenditure (in absolute terms and as a share of the gross domestic product and overall budget) indicate government commitment to health [11].

For West African countries to move towards the target of UHC, healthcare systems need to be adequately strengthened. Out-of-pocket payment has driven many people below the poverty line [3, 16]. This is not in line with the goal of UHC, as access to needed health services should not expose individuals to financial hardship. A key element of the UHC includes financial protection from the costs of ill health and access to and use of needed health services within a country. Thus, reducing the reliance on out-of-pocket payments for healthcare is important for financial protection [16]. This makes the government’s health expenditure critical to the achievement of the objectives of UHC, as a considerable increase in government expenditure on health will drastically reduce out-of-pocket payments and increase the financial protection of the populace.

West Africa is characterized by much diversity in terms of demographics, and health outcomes among others (see Table 1). These factors have not only contributed to the differences in the health status of the region’s diverse populations but also to the diverse nature of its health systems. Therefore, this study aimed to assess health priorities using the government health expenditure and the general government expenditure in the Economic Community of West African States (ECOWAS) from 2010 to 2018.

**Table 1:** Total population and selected health indicators in ECOWAS. Source: Authors compilation from Global Health Observatory Database. Accessed link: https://www.who.int/data/gho/data/indicators Accessed 29 Sept 2021

Country/Indicator	Total population (in thousands)—2016	Life expectancy at birth (years)—2019	Under-five mortality rate (probability of dying by age 5 per 1000 live births)—2019	Maternal mortality ratio per 100,000 live births—2017	Total NCDs age-standardized mortality rate (per 100,000 population)—2019	Out-of-pocket expenditure as percentage of current health expenditure (%)—2018
Benin	10,872	63.43	90.29	397	569.8	44.55
Ghana	28,207	66.28	46.16	308	551.1	37.69
Nigeria	185,990	62.62	117.2	917	512.3	76.60
Burkina Faso	18,646	62.70	87.54	320	566.3	35.83
Cabo Verde	540	74.03	14.86	58	405.8	27.96
Cote d’Ivoire	23,696	62.92	77.50	617	527.5	39.43
The Gambia	2039	65.47	51.72	597	553.7	29.34
Liberia	4614	64.08	84.62	661	484.3	41.79
Togo	7606	64.27	66.90	396	558.4	56.32
Mali	17,995	62.80	94.04	562	604.1	33.90
Niger	20,673	63.29	80.37	509	575.8	48.79
Senegal	15,412	68.58	45.31	315	529.5	55.89
Guinea	12,396	61.01	98.8	576	632.1	60.62
Sierra Leone	7396	60.77	109.2	1120	631.8	44.78
Guinea-Bissau	1816	60.22	78.47	667	604.8	74.48

## Method

**Fig. 1:**
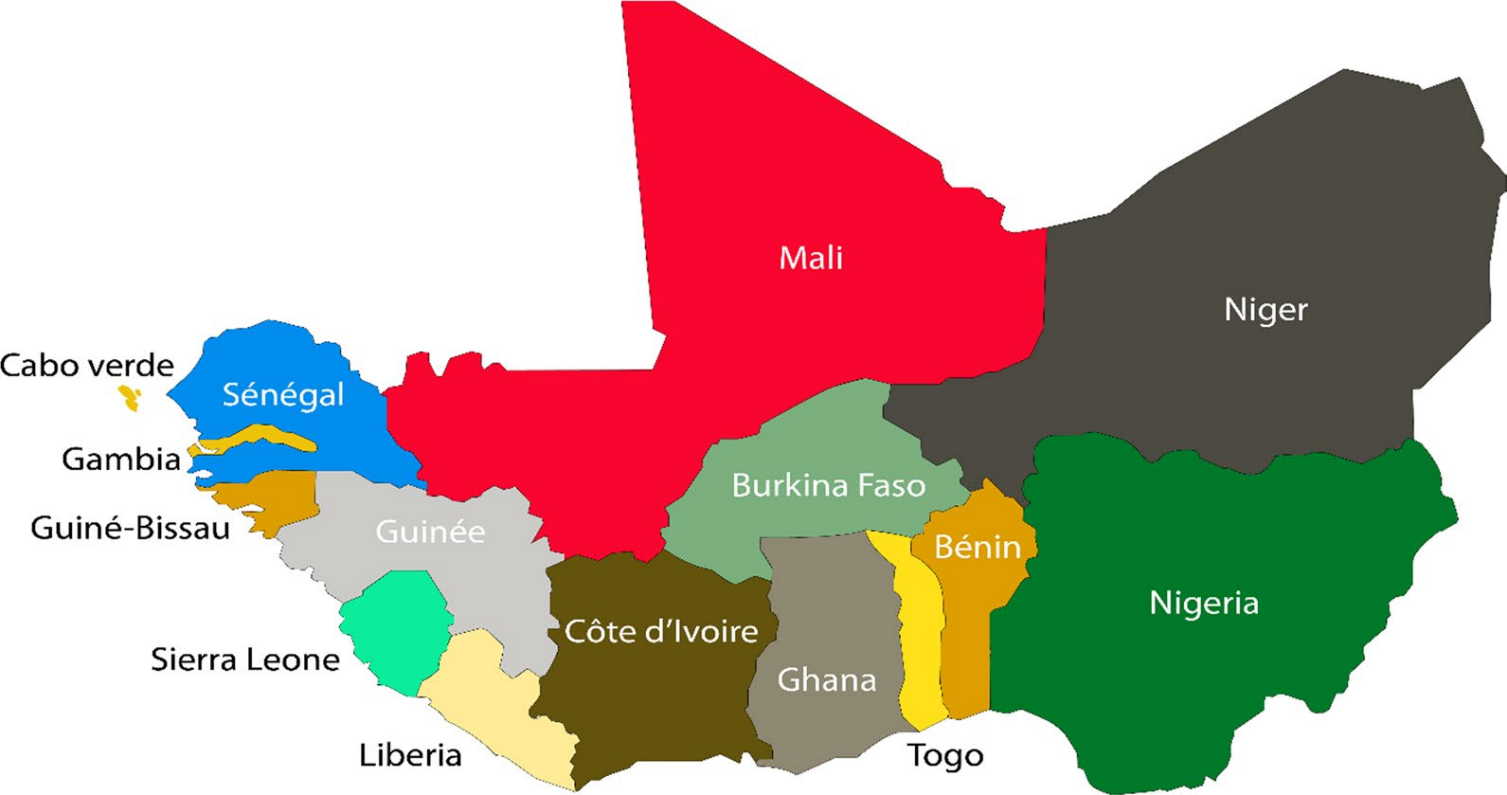
Illustration showing ECOWAS member states

We extracted and analysed data from 2010 to 2018 on health priority (the share of domestic general government health expenditures of general government expenditure) in the WHO’s Global Health Expenditure Database (https://apps.who.int/nha/database/country_profile/Index/en) across the 15 members of the ECOWAS (Benin, Burkina Faso, Cabo Verde, Cote d’Ivoire, The Gambia, Ghana, Guinea, Guinea-Bissau, Liberia, Mali, Niger, Nigeria, Senegal, Sierra Leone, and Togo) (see Fig. 1). The data available from the WHO Global Health Expenditure database are reported by country governments using the framework of System of Health Accounts 2011. The database provides internationally comparable data on health spending for close to 190 countries including West African countries from 2000 to 2018. The database is open access, and it supports the goal of UHC by assisting in monitoring the availability of resources for health and the extent to which they are used efficiently and equitably. We considered the years 2010–2018, because it covers certain parts of the Millennium Development Goals (MDGs) and Sustainable Development Goals (SDGs) era. This provides an insight into the progress made in improving health across West Africa over time.

We also identified that the ideal health priority index should be equal to a minimum of 15%, considering the formula = (government health expenditure over government total expenditure) × 100. The minimum government health expenditure should be 15% [3]. That implies the net government expenditure should be set at 100% (minimum of 15% to health; minimum of 85% to other areas). Then, health priority = (15%/100%) × 100 = 15%. We summarized health priorities across 15 countries in ECOWAS from 2010 to 2018 and compared the average health priorities to the minimum of an ideal health priority index of 15%. The data are presented using descriptive statistics.

## Results

Table 2 shows health priorities across West African countries from 2010 to 2018. Our findings revealed that Ghana has the highest average health priority (8.29%), while Guinea has the lowest average health priority (3.05%) in the West African region within the period of our analysis.

**Table 2:** Assessment of health priorities in The Economic Community of West African States from 2010 to 2018

Country	Health priority (%)									Average health priorities from 2010–2018
	2010	2011	2012	2013	2014	2015	2016	2017	2018	
Benin	5.15	5.40	5.25	5.13	4.04	3.23	3.73	4.58	2.96	4.39
Ghana	11.91	12.09	8.97	8.64	6.72	8.56	6.54	6.04	6.42	8.43
Nigeria	2.69	2.76	3.87	3.66	3.53	5.32	5.00	4.44	4.44	3.96
Burkina Faso	6.03	6.59	4.70	6.38	7.77	7.15	11.03	9.99	8.78	7.60
Cabo Verde	7.23	8.61	8.86	9.03	9.98	10.09	10.39	10.39	10.39	8.29
Cote d’Ivoire	4.06	4.39	4.62	4.59	5.11	4.75	4.82	5.10	5.07	4.72
The Gambia	6.13	6.13	6.13	6.13	5.75	5.10	6.23	4.38	4.38	5.59
Liberia	3.17	5.05	2.84	1.03	2.51	3.25	3.90	4.20	5.23	3.46
Togo	7.45	5.67	6.11	5.01	5.07	4.16	4.26	4.26	4.26	5.14
Mali	3.30	2.94	2.66	4.13	4.45	4.39	5.43	5.43	5.43	4.24
Niger	8.70	8.94	6.25	6.56	5.40	4.59	5.69	9.66	8.35	7.13
Senegal	5.35	5.63	5.31	5.30	4.68	4.69	4.46	4.26	4.26	4.88
Guinea	1.77	3.18	2.57	2.62	3.03	1.98	4.11	4.11	4.11	3.05
Sierra Leone	6.29	4.13	4.68	5.15	7.58	7.91	7.91	7.91	7.25	6.53
Guinea-Bissau	5.94	2.87	4.20	4.72	2.89	2.78	2.68	3.01	3.02	3.56

Source: Authors’ compilation from World Health Organization Global Health Expenditure Database

**Fig. 2:**
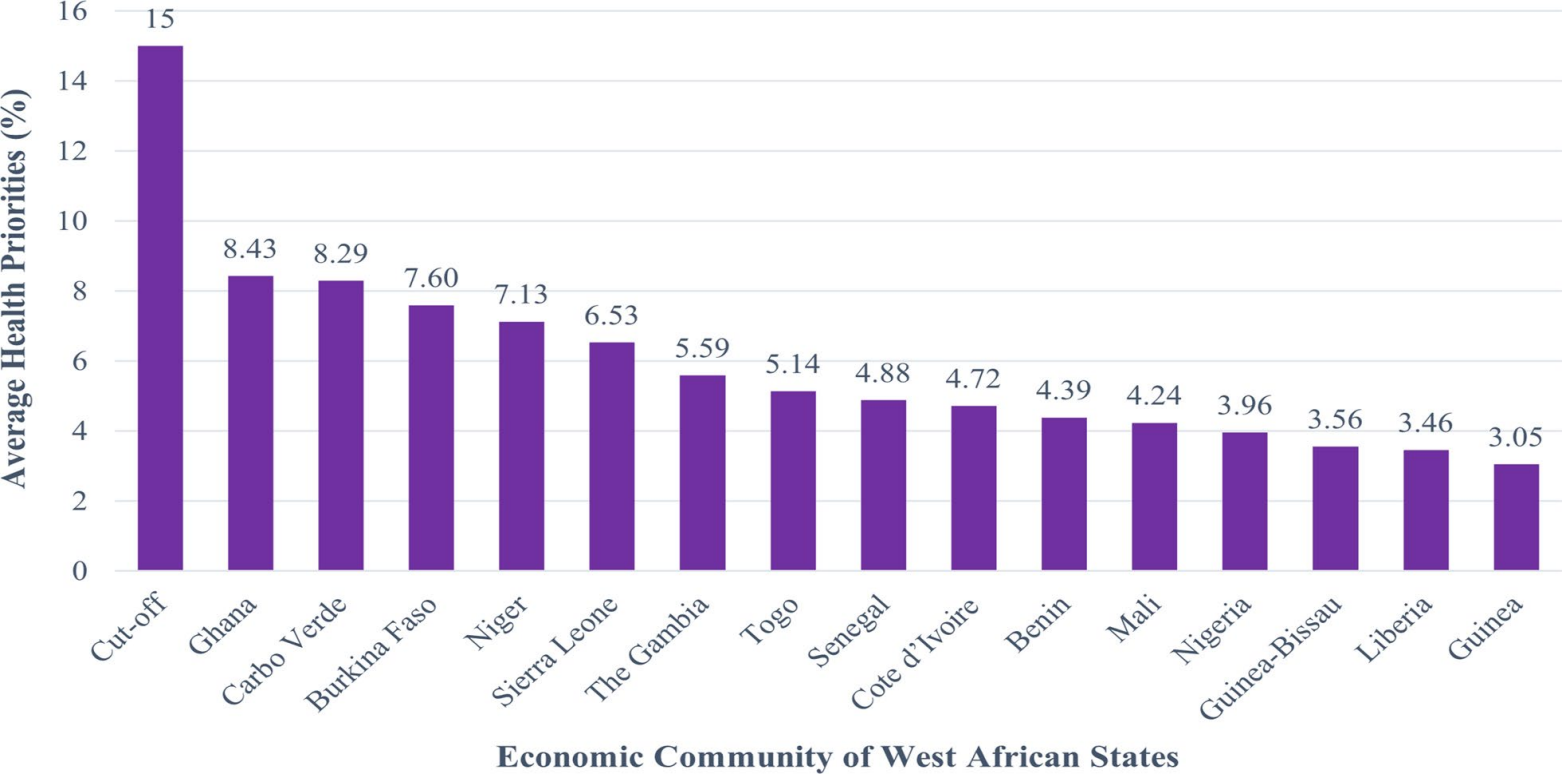
Plot of the average health priorities in the Economic Community of West African States including cutoff point of at least 15%

Figure 2 also reveals that no West African countries beat the cutoff of a minimum of 15% health priority index.

## Discussion

### State of health priority in West Africa

Amidst the increasing cost of health services globally and escalating prevalence of endemic and epidemic diseases plaguing the West African subcontinent, the health priority of ECOWAS countries remains inadequate. Expressed in percentage as the government expenditure on health over the total expenditure, the health priority of each country is used as a tool to determine the level of prioritization of health by the governments through their budgetary allocations. Over the years, the majority of West African countries have not met the African head of state commitment to allocate at least 15% of the annual budget to health sector. Quite confoundingly, health priority in a sizeable number of countries saw an appreciable reduction over the years as evident by Ghana, Togo, and Guinea-Bissau. The mean value for health priorities averaged by all the countries is a paltry 5.4%. For a substantive number of countries, the total health expenditure is far less than the bare minimum of $34 per person per year recommended by the WHO Macroeconomics and Health [17].

Healthcare is not a luxury but rather a necessity and this critical underfunding of health correlates with negative life expectancy and economic growth retardation profoundly battling the West African countries [18]. All these regions are faced with numerous health problems including healthcare workers shortages, poorly equipped primary healthcare facilities, incessant brain drain, political flux, drug shortages [19], widespread use of fake drugs, high maternal and child mortalities, the burden of infectious diseases, and poverty and low standard of living [20]. All these stem from a shortage of health expenditure exacerbated by the inefficient use of existing resources, lack of effective social protection mechanism to ensure equitable distribution of healthcare, gross incapability to implement national policies, nepotism, and deficient political moxie.

Primarily, the lack of investment by the government on the healthcare system has also resulted into weak insurance systems on the continent [3, 6]. In many West African Countries, the out-of-pocket payment resulting in catastrophic spending is worrisome, even though there are various public and private insurance establishments [3]. This is concerning, because uninsured patients are at the mercy of an inefficient health system indicating an overall lack of health priority by the government.

### Reason for this state of health priority

Health indices in West Africa are worrisomely by far the lowest in the world and the health expenditure is abysmally discouraging. It becomes expedient to analyze the reason for this to derive the implications on UHC. Micah and colleagues [21] considered seven major factors influencing health budgetary allocation, namely: inadequate national income, profligate general government expenditure on non-health-related sectors, inadequate external revenue and assistance for health, limited government tax revenue caused by large informal sectors vis-à-vis small formal sectors, profound corruption in the public sector, unfavorable time trend, and huge population structure. At the heart of these factors lie government low health financing; low investment in other sectors that influence social determinants of health including water and sanitation, food security, and housing; preponderant un-pooled out-of-pocket spending; ineffectual health insurance schemes; inefficient health programme integration; and poor health strengthening systems approach.

### Implications for Universal Health Coverage

Achieving UHC is a major target every country in the world aimed to attain when adopting the SDGs in 2015. This implies that all individuals and communities will have access to healthcare without suffering financial hardship, as such promote health equity [16]. This concept, as laudable as it is, is heavily dependent on the healthcare expenditure by the government of every country. With the inadequate health priority as observed in the West African countries, the core objectives of UHC become encumbered with challenges. One of such objectives is financial protection which implies that the cost of assessing medical services should not impact negatively on the recipients. This is to ensure that all individuals have sufficient access to healthcare needs without significant out-of-pocket spending to obtain healthcare. This is usually achieved by risk pooling through tax-funded or social health insurance schemes [3, 22]. In most West African countries, un-pooled sources include out-of-pocket spending in the forms of payment for medical products and services accounts for up to 30% to 70% of total health expenditure [3]. The reliance on out-of-pocket spending significantly impedes access to healthcare and predisposes the population to impoverishment [22, 23].

Another important aim of UHC is to ensure equitable access to health services through adequate provision of quality preventive and treatment healthcare services as at when due without denial in access due to lack of healthcare facility, supplies, and manpower [3]. In addition, from every indication, West African countries’ low health prioritization mount a huge roadblock against this aim as evident by its characteristic deficient health facilities, low drug availabilities, heavy shortage of healthcare practitioners, dilapidated health institutions, and enormous vulnerable population, resulting in very low confidence in the struggle to achieve UHC.

### Recommendations

Having established the dire state of health priority in West African countries, we identify various means of ameliorating this swathe of problems. These include but are not limited to improvement of the government’s commitment to providing adequate and necessary healthcare through the strengthening of their willpower and imperative to fill the huge gaps in healthcare provision and financing; investment in strengthening the region’s primary healthcare systems; provision of incentive to private health sectors to alleviate their huge cost of health services; implementation of strong monitoring framework that ensures prudent utilization of government expenditure on health and to curb incompetence and corruption in budgeting, planning, accounting, auditing, monitoring and evaluation at all levels; a capacity building that ensures the development of prepaid health financing systems including social health insurance, community health insurance, and tax-funded systems to cushion households against catastrophic and impending pauperization.

The indicators of health in West Africa countries are abysmal. We have a picture of a region plagued with diverse health problems and concomitant morbidities and mortalities. Despite the availability of policies and initiatives steered towards accentuating governments' priority on health through budgetary allocations, there has been an underwhelming outcome on health indices in the region. This is largely a function of the government’s failure to allocate an appreciable quota of their budget on health. Consequently, the proportion of individuals who can afford the astronomical cost of healthcare continue to spend hugely to obtain even the basic healthcare services at a suboptimal level, while the proportion of those who cannot afford healthcare continues to escalate. This directly contradicts the aims and objectives of the UHC and SDGs. Thus, it becomes imperative to anatomize the crux of this perpetual problem. Consensus materialized on several key actions that must be taken to allay impending negative health implications on the West African subcontinent. Every country must develop a comprehensive health financing strategy with a clear roadmap for attaining the UHC vision. Countries must make concerted efforts to abide by the policies that ensure that at least 15% of their annual budget is allocated to health. A monitoring framework that regulates government allocation and ensures judicious utilization of government expenditure must be reinstated to curb excesses, defiance, and libertine misuse of government resources. Only then will the West Africa region liberate itself from the constraints of ill-health and begin the journey towards development on par with contemporaneous regions of the world.

## Conclusion

This study emphasized the need for West African governments and other relevant stakeholders to prioritize health in their political agenda towards achieving UHC. It is also important that every country in the region develop a comprehensive health financing strategy with a clear roadmap for attaining the UHC vision.

## Data Availability

Not applicable.
